# Distribution characteristics of integrons and correlation analysis of antibiotic resistance in *Aeromonas hydrophila*

**DOI:** 10.3389/fcimb.2025.1709447

**Published:** 2026-01-16

**Authors:** Taotao Yang, Xuedan Qiu, Wenjun Lu, Jianqiang Xu, Yu Ye, Qiaoping Wu, Qingcao Li

**Affiliations:** 1 Office of Good Clinical Practice, The Affiliated Li Huili Hospital of Ningbo University, Ningbo, China; 2 Department of Clinical Laboratory, The Affiliated Li Huili Hospital of Ningbo University, Ningbo, China; 3 Intensive Care Unit, The Affiliated Li Huili Hospital of Ningbo University, Ningbo, China; 4 Department of Clinical Laboratory, Yuyao Third People’s Hospital, Ningbo, China; 5 State Key Laboratory for Diagnosis and Treatment of Severe Zoonostic Infectious Disease, Wuhan, China

**Keywords:** *Aeromonas hydrophila*, ERIC-PCR, homology, integron, resistance gene

## Abstract

**Objective:**

This study aims to investigate the distribution of integrons in clinically isolated *Aeromonas hydrophila* from our hospital, as well as the correlation between antibiotic resistance, resistance genes and integrons carried by *Aeromonas hydrophila.* Through molecular characterization of integrons and associated resistance gene cassettes, we seek to gain an in-depth understanding of the role of integrons in mediating multidrug resistance in *Aeromonas hydrophila*, thereby providing a basis for more effective infection control strategies.

**Methods:**

We collected 80 strains of clinically isolated *Aeromonas hydrophila* from January 2021 to December 2024 and performed antimicrobial susceptibility testing on them. Polymerase chain reaction (PCR) was used to screen these strains for class 1, 2, and 3 integrons, resistance genes, and virulence factors. Subsequently, the variable regions of integron-positive strains were amplified and sequenced. An analysis was conducted to assess the correlation among bacterial drug resistance, resistance genes, virulence genes, and integrons. Additionally, enterobacterial repetitive intergenic consensus PCR (ERIC-PCR) was employed to assess the clonal relatedness of integron-positive strains.

**Results:**

Among the 80 clinical isolates, 16 (20.0%) were positive for class 1 integrons, while no class 2 or 3 integrons were detected. Amplification of the variable regions of class 1 integrons identified four resistance gene cassettes, predominantly *catB8* and *aadA1*. The integron-positive strains exhibited significantly higher resistance rates to ceftazidime, gentamicin, imipenem, trimethoprim-sulfamethoxazole, and amikacin compared to integron-negative strains (*P < 0.05*). Additionally,The detection rate of β-lactamase gene *MOX* in drug-resistant genes was the highest, accounting for 62.5%, and the detection rate of virulence gene *ast* was the highest, accounting for 65.0%. ERIC-PCR typing classified the 16 integron-positive strains into seven genotypes, with type C being the most predominant. The *catB8*-*aadA1* gene cassette was mostly found in type C strains, which were predominantly isolated from the Hepatobiliary and Pancreatic Surgery Department.

**Conclusion:**

Our study reveals that class 1 integrons are the predominant type carried by *Aeromonas hydrophila* clinical isolates in our hospital. The aminoglycoside resistance gene *aadA1* and the chloramphenicol resistance gene *catB8*, identified within the variable regions of these integrons, are directly associated with corresponding antibiotic resistance phenotypes. Notably, integron-positive strains displayed high clonal similarity, with the dominant ERIC genotype C indicating potential clonal dissemination within the hospital setting. These findings suggest that integrons, along with their carried resistance gene cassettes, could serve as useful molecular markers for epidemiological surveillance of multidrug-resistant *Aeromonas hydrophila*. Implementing routine screening for integrons and associated resistance genes in high-risk wards, such as hepatobiliary surgery, could enhance targeted infection control measures and help prevent the spread of resistant clones, including those of emerging pathogens like *A. hydrophila*.

## 1 Introduction

*Aeromonas hydrophila*, a Gram-negative bacterium widely distributed in natural environments, is not only a significant pathogen in aquaculture—causing severe diseases such as septicemia, ulcerative lesions, and hemorrhagic enteritis in various freshwater fish, often leading to mass mortality—but also a common pathogen in reptiles, amphibians, and poultry (Salifu et al., 2023). In terms of human health, this bacterium holds significant public health importance. It can not only cause acute gastroenteritis through the consumption of contaminated aquatic products but also poses a risk of invading the bloodstream via the intestinal mucosa, leading to septicemia (Harandou et al., 2025). Furthermore, *Aeromonas hydrophila* can induce various clinical conditions such as respiratory infections, skin and soft tissue infections, as well as bloodstream infections, presenting a particularly severe threat to immunocompromised individuals (Srivastava et al., 2023). In recent years, its isolation rate from clinical specimens has been progressively increasing, establishing it as an important nosocomial pathogen (Kabwe et al., 2020). The widespread use of antibiotics has led to escalating drug resistance in *Aeromonas hydrophila* (Ren et al., 2019), posing significant therapeutic challenges. While current research primarily focuses on phenotypic resistance patterns, limited attention has been paid to integrons’ role in resistance dissemination (Pourmohsen et al., 2023). As crucial mobile genetic elements, integrons play a pivotal role in horizontal transfer of resistance genes among bacteria (Ghaly et al., 2020). Understanding their relationship with *Aeromonas hydrophila* resistance is therefore critical for clinical management and infection control. Through comprehensive analysis combining antimicrobial susceptibility testing, detection of integrons/resistance/virulence genes, variable region sequencing, and ERIC-PCR genotyping, this study aims to provide the first systematic evaluation of the interplay between resistance profiles, genetic determinants, and integrons in clinical *Aeromonas hydrophila* isolates. Specifically, we seek to investigate the distribution and molecular characteristics of integrons, analyze their correlation with antibiotic resistance phenotypes and resistance genes, and elucidate their role in mediating multidrug resistance in these clinical isolates. Therefore, elucidating the prevalence and role of integrons in mediating multidrug resistance in *Aeromonas hydrophila* is not only crucial for understanding its resistance evolution but also has direct implications for guiding clinical infection control and antibiotic stewardship. Our findings establish an evidence base for targeted infection control measures, particularly in preventing the spread of high-risk clones within healthcare settings.

## 2 Materials and methods

### 2.1 Bacterial strains and specimen source

This investigation utilized 80 clinically isolated, non-duplicate strains of *Aeromonas hydrophila* collected from the Affiliated Li Huili Hospital of Ningbo University between January 2021 and December 2024. The study protocol received ethical approval from the Institutional Review Board of The Affiliated Li Huili Hospital of Ningbo University (Approval No. KY2024SL497-01). For quality assurance purposes, *Pseudomonas aeruginosa* ATCC 27853 served as the reference strain for antimicrobial susceptibility testing validation. Control strains included *Escherichia coli* DH5α as the negative control for all three classes of integrons, while *Proteus mirabilis* 47437 and *Serratia marcescens* 37586 functioned as positive controls for class 1/2 and class 3 integrons, respectively. All reference microorganisms were obtained from the National Center for Clinical Laboratories under the National Health Commission’s supervision. The experimental design ensured standardized comparison through these validated control strains while maintaining methodological rigor in integron detection and characterization.

### 2.2 Identification of bacterial strains and antibiotic resistance testing

Bacterial identification and antimicrobial susceptibility testing were performed strictly following the standard operating procedures of the VITEK 2 automated microbial identification system (bioMérieux, France). Single colonies of test strains were picked and suspended in sterile saline (4.5% NaCl) to achieve a 0.5 McFarland standard turbidity. The prepared suspensions were then loaded into VITEK 2 identification cards (GN) and antimicrobial susceptibility testing cards (N13) for species identification and drug sensitivity analysis. All antibiotic susceptibility interpretations were made according to the latest CLSI guidelines (2024 edition). Comprehensive quality control measures were implemented throughout the testing process to ensure the reliability and accuracy of results.

### 2.3 DeoxyriboNucleic acid template preparation, screening for integrons and variable regions

All DNA templates were prepared using a constant-temperature metal bath. For PCR amplification, Premix Taq™ DNA polymerase (TaKaRa, Japan) was used to detect class 1, 2, and 3 integrons, while LA Taq DNA polymerase (TaKaRa, Japan) was employed to amplify the variable regions of integrons. The specific primer sequences used are listed in **Supplementary Table 1**. The above experimental method, which included appropriate negative and positive controls in each batch following established methodologies (Lu et al., 2022; Qiu et al., 2024), has been widely applied in integron-related detection and is widely recognized in the industry (Sun et al., 2022; Zuberi and Sillo, 2022; Wang et al., 2023).

### 2.4 Sequencing of integrase and variable region

The amplification products of integrons and variable regions showing distinct bands on electrophoresis indicate the presence of gene cassettes and corresponding sequences. The PCR products from strains with positive electrophoretic bands were sent to BGI (Beijing Genomics Institute) for sequencing analysis. The obtained sequencing results were aligned using BLAST to determine the integron type and gene cassettes within the variable region, with the integron type being specifically identified based on the sequencing results of the integrase gene. Sequencing and BLAST alignment were essential to definitively confirm the identity of the amplified variable regions and their carried gene cassettes, as PCR amplification alone, despite rigorous controls, cannot exclude non-specific products or conclusively determine sequence composition.

### 2.5 Screening of antibiotic resistance genes and whole-genome sequencing

To identify the presence of antibiotic resistance genes and virulence genes, we performed PCR amplification using specific primers targeting resistance genes such as β-lactamase genes, extended-spectrum β-lactamase (ESBL) genes, and carbapenemase genes, as well as virulence genes (see **Supplementary Table 1** for relevant primers), to screen for the existence of these genes in the template DNA of bacterial isolates. The PCR mixture (total volume: 25 µL) contained 1 µL of genomic DNA template, 1 µL of each primer, 12.5 µL of Premix rTaq PCR solution (TaKaRa, Japan), and 9.5 µL of distilled water. Amplification was performed using an ABI Veriti thermal cycler (Applied Biosystems, Singapore), with reaction conditions adjusted according to the referenced protocols. The PCR products were subsequently confirmed by electrophoresis and sequencing, and the resulting sequencing results were matched using BLAST. Based on the distribution of integrons, drug resistance genes, and the antimicrobial susceptibility profile, a representative bacterial strain was selected for next-generation sequencing. Genomic DNA was extracted and submitted to MajorBio (Shanghai, China) for sequencing on the Illumina NovaSeq 6000 platform (San Diego, CA, USA). Raw sequencing data were quality-assessed using FastQC (v0.11.9), and low-quality reads along with adapter sequences were trimmed with Trimmomatic (v0.39) under the following parameters: sliding window 4:20, minimum read length 50. Clean data were *de novo* assembled with SPAdes (v3.15.4) using default settings to produce a draft genome. The quality of the genome assembly was evaluated with QUAST (v5.0.2).

### 2.6 ERIC-PCR homogeneity testing

The ERIC-PCR reaction mixture (total volume: 25 μL) consisted of 16.25 μL deionized water, 6.75 μL RTaq DNA polymerase reaction buffer (containing dATP, dTTP, dCTP, and dGTP), 1 μL ERIC2 primer, and 1 μL template DNA. The amplification protocol included: initial denaturation at 94 °C for 4 min; 40 cycles of denaturation at 94 °C for 40 s, annealing at 40 °C for 1 min, and extension at 72 °C for 5 min; followed by a final extension at 72 °C for 10 min. Amplified products were electrophoresed, and bands at identical positions were considered to represent the same genotype. Strain similarity was analyzed using the UPGMA method (unweighted pair group method with arithmetic mean) and Dice similarity coefficient in NTsys 2.10e software.

### 2.7 Statistical analysis

Statistical analysis was performed using SPSS software (version 25.0). The chi-square test (χ² test) was applied to compare antibiotic resistance rates and resistance gene distributions between integron-positive and integron-negative strains. A P-value <0.05 was considered statistically significant.

For the ERIC-PCR genotyping of integron-positive strains, the banding patterns obtained through electrophoresis were analyzed by converting them into a binary matrix based on the presence or absence of bands at each position. Genetic similarity between strains was calculated using the Dice similarity coefficient. Subsequently, the unweighted pair group method with arithmetic mean (UPGMA) was applied to construct a dendrogram reflecting the phylogenetic relationships among the strains.

### 2.8 Data availability

The complete genome sequences of 1 strains of *Aeromonas hydrophila* have been deposited to National Center for Biotechnology Information (NCBI) under the BioProject number PRJNA1321511.

## 3 Results

### 3.1 Analysis of the distribution characteristics of clinical isolates

A total of 80 clinical isolates of *Aeromonas hydrophila* were collected in this study. The distribution of specimen sources was as follows: bile (28 isolates, 35.0%), blood (13 isolates, 16.3%), drainage fluid (12 isolates, 15.0%), sputum (12 isolates, 15.0%), urine (6 isolates, 7.5%), secretion (4 isolates, 5.0%), and others (5 isolates, 6.2%). In terms of departmental sources, the Hepatobiliary and Pancreatic Surgery Department contributed the highest number of isolates (25 isolates, 31.3%), followed by the Hematology Department (13 isolates, 16.3%), Gastrointestinal Surgery Department (8 isolates, 10.0%), Gastroenterology Department (7 isolates, 8.8%), Minimally Invasive Abdominal Surgery Department (6 isolates, 7.5%), and Urology Department (3 isolates, 3.8%). The remaining 18 isolates (22.5%) were obtained from other departments.

### 3.2 The results of integrons screening for clinical strains

**Figure 1:**
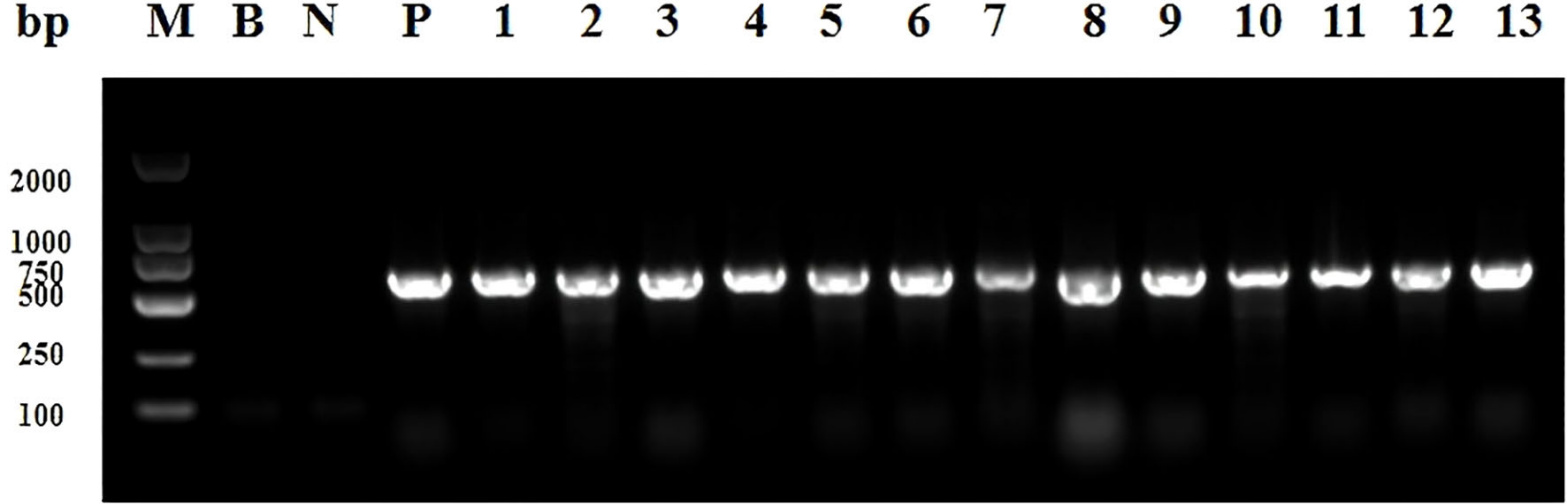
Electrophoresis image of PCR products for screening class 1 integrons in partial experimental bacterial strains. Lane M represents the DNA Marker; Lane B is the blank control; Lane N represents the negative control; Lane P corresponds to the positive control; Lanes 1 to 13 display the results of selected subject-derived bacterial strains positive for Class 1 integrons, specifically strains 2, 12, 15, 32, 33, 34, 40, 47, 48, 61, 62, 63, and 68. A positive band at 615 bp indicates the presence of class 1 integron.

Among the 80 clinical isolates, 16 (20.0%) tested positive for class 1 integrons, while no class 2 or class 3 integrons were detected. The electrophoretic results of class 1 integron amplification products from selected clinical isolates are shown in **Figure 1**.

### 3.3 The detection results of the variable region resistance gene cassette

**Figure 2:**
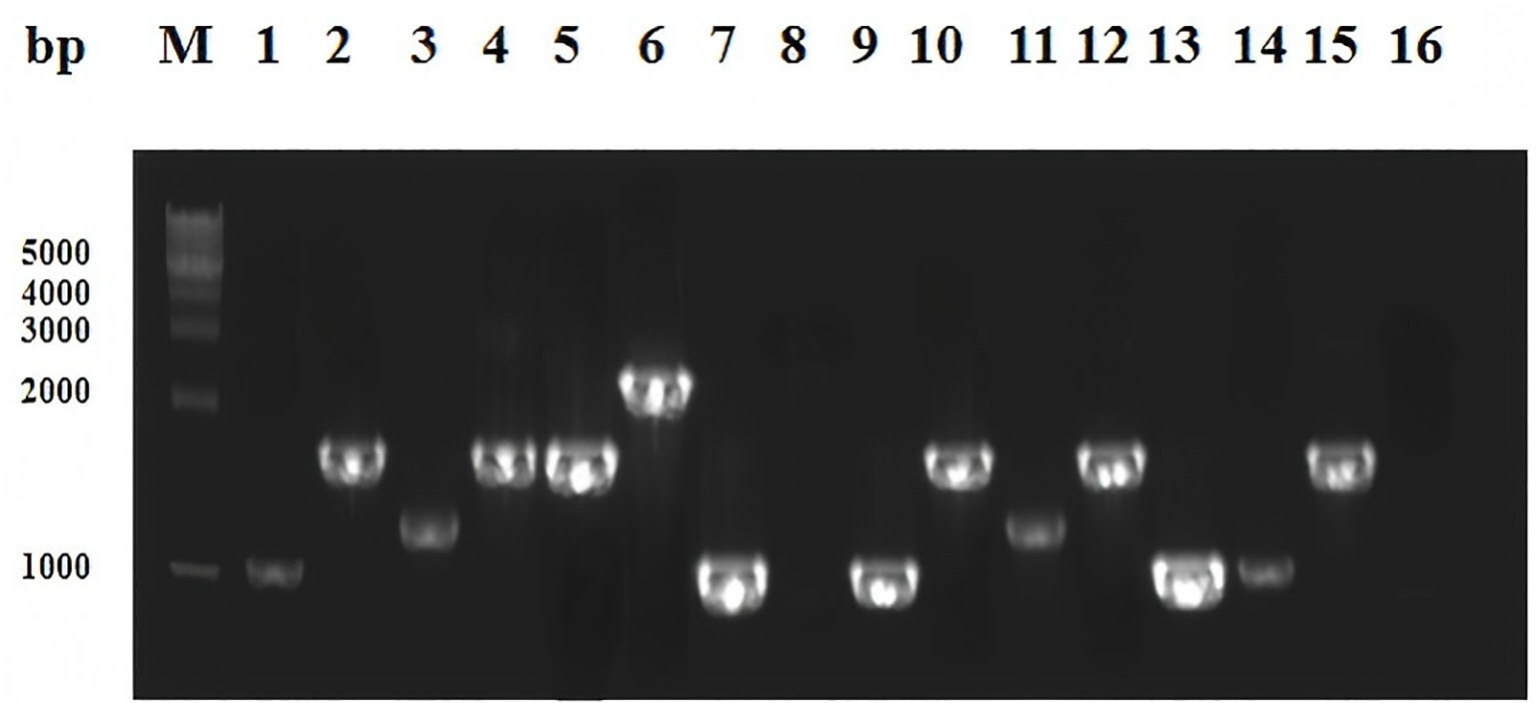
Electrophoresis image of PCR products for variable region of partial class 1 integrons. Lane M represents the DNA marker, while lanes 1 to 16 correspond to class 1 integron-positive strains, specifically strains 2, 12, 15, 32, 33, 34, 40, 47, 48, 61, 62, 63, 68, 69, 71, and 72. The integron variable regions at the same band position are considered to be of the same size.

Electrophoresis analysis of the variable regions from 80 class 1 integron-positive isolates revealed visible amplification bands in 14 strains. These bands primarily corresponded to four distinct sizes, approximately 1 kb, 1.1 kb, 1.5 kb, and 2.0 kb. Two strains showed no amplification products. The electrophoretic results of the variable region amplification are presented in **Figure 2**.

### 3.4 The sequencing results of the variable region amplification products

The amplified products of class 1 integron variable regions were sequenced and analyzed by BLAST alignment. Four antibiotic resistance gene cassettes were identified in these variable regions, with *catB8* (Chloramphenicol resistance gene) and *aadA1* (Aminoglycoside drug resistance gene) being the predominant types (**Table 1**).

**Table 1:** Distribution of gene cassettes in class 1 integron-positive aeromonas hydrophila.

Number	IntI1	Variable region of class 1 integron
6	+	*catB8-aadA1*
5	+	*aadA4a*
2	+	*dfrA1-aadA1*
1	+	*arr-2-aacA4-dfrA1-orfc*
2	+	ND

IntI1: Class 1 Integron, ND: no PCR product detected, *catB8:* chloramphenicol acetyltransferase*. aadA:* aminoglycoside adenylvotransferase*. dfrA*: trimethoprim resistance gene, *arr*: rifampin resistance, *aacA*: aminoglycoside acetyltransferase, *orfc*: An open reading frame (ORF) of unknown function. Similar to *ybeA*, *ybfA*, etc., its potential role in resistance or other cellular processes remains to be determined.

### 3.5 Prevalence of antimicrobial resistance and virulence genes

**Figure 3:**
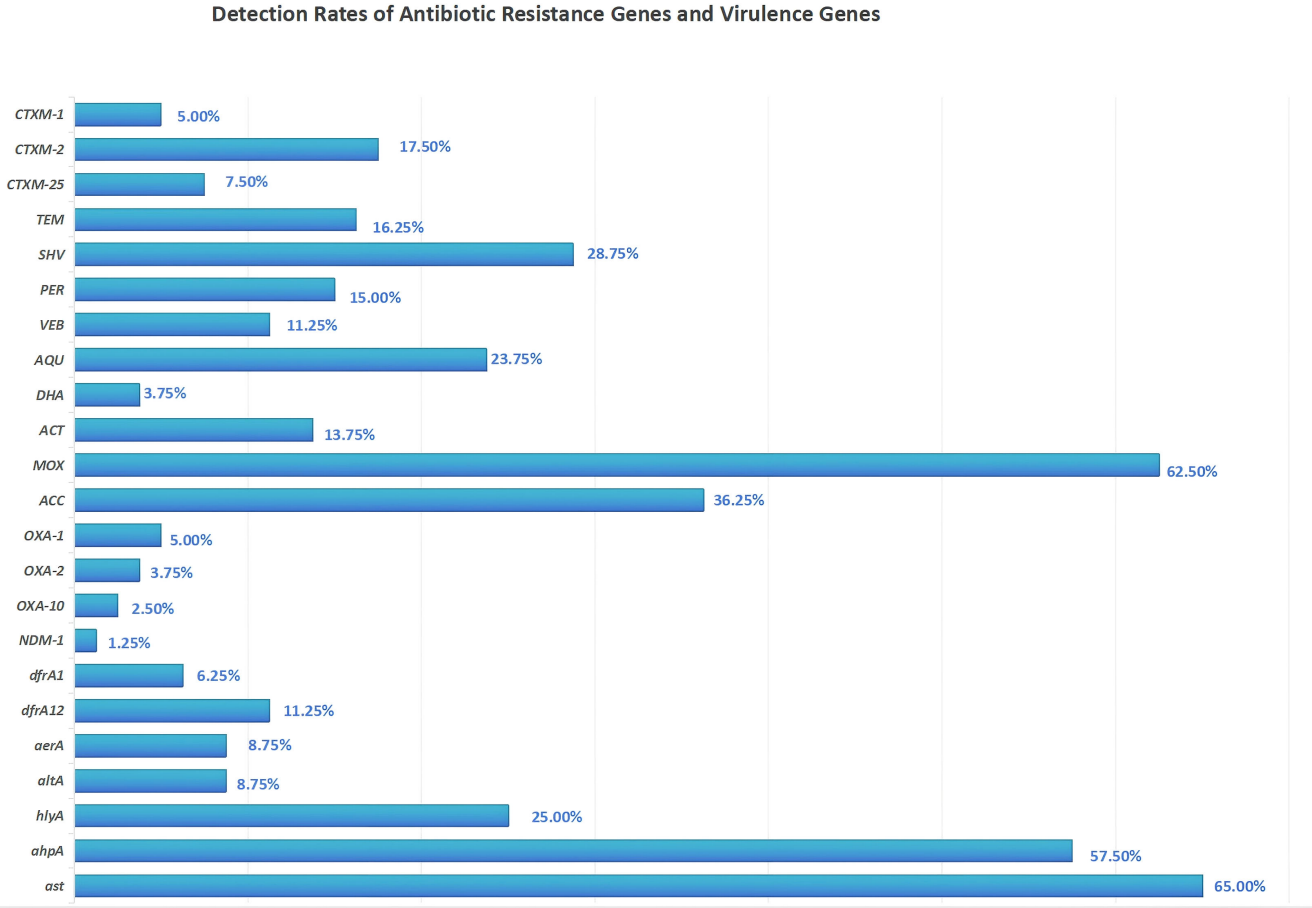
Detection rates of antibiotic resistance genes and virulence genes.

In this study, a total of 80 bacterial isolates were screened for the presence of antibiotic resistance genes and virulence genes. The results revealed that among the β-lactamase genes, *MOX* demonstrated the highest detection rate (50 isolates, 62.5%), followed by *SHV* (23 isolates, 28.8%) and *ACC* (29 isolates, 36.3%). For *ESBL* genes, *CTXM-2* (14 isolates, 17.5%) and *TEM* (13 isolates, 16.3%) were relatively common, while *CTXM-8* and *CTXM-9* were not detected. Additionally, the carbapenemase gene *NDM-1* was identified in only one isolate (1.3%). Among non-β-lactam resistance genes, the aminoglycoside resistance gene *AQU* (19 isolates, 23.8%) and the sulfonamide resistance gene *dfrA12* (9 isolates, 11.3%) were notable. Regarding virulence genes, ast showed the highest prevalence (52 isolates, 65.0%), followed by *ahpA* (46 isolates, 57.5%) and *hlyA* (20 isolates, 25.0%). In contrast, *aerA* and *altA* were detected at relatively low frequencies (7 isolates each, 8.8%). It is worth noting that no significant difference (P > 0.05) was observed in the carriage of antibiotic resistance or virulence genes between integron-positive and integron-negative strains. The detailed distribution of these genes is presented in **Figure 3**.

### 3.6 The relationship between bacterial strain resistance and integrons

*Aeromonas hydrophila* exhibited high resistance rates to ceftazidime, imipenem, and trimethoprim-sulfamethoxazole. Notably, integron-positive strains demonstrated significantly higher resistance to ceftazidime, gentamicin, imipenem, trimethoprim-sulfamethoxazole, and amikacin compared to integron-negative strains (*P<0.05*). The antibiotic resistance profiles, categorized by integron presence, are summarized in **Table 2**.

**Table 2:** Comparison of antibiotic resistance rates between integrase-positive and integrase-negative strains.

Antibiotics	Integrase-positive strains (n=16)		Integrase-negative strains (n=64)		*P* value
	Number	Resistance rate (%)	Number	Resistance rate (%)	
Aztreonam	2	12.5	5	7.8	0.62
Ceftazidime	14	87.5	16	25.0	<0.05
Ciprofloxacin	2	12.5	3	4.7	0.26
Cefepime	2	12.5	1	1.6	0.10
Gentamicin	5	31.5	0	0.0	<0.05
Imipenem	8	50.0	8	12.5	<0.05
Levofloxacin	2	12.5	1	1.6	0.10
Sulfamethoxazole-Trimethoprim	13	81.3	1	1.6	<0.05
Piperacillin-Tazobactam	2	12.5	13	20.3	0.72
Amikacin	2	12.5	0	0.0	<0.05

### 3.7 Results of second-generation sequencing

**Figure 4:**
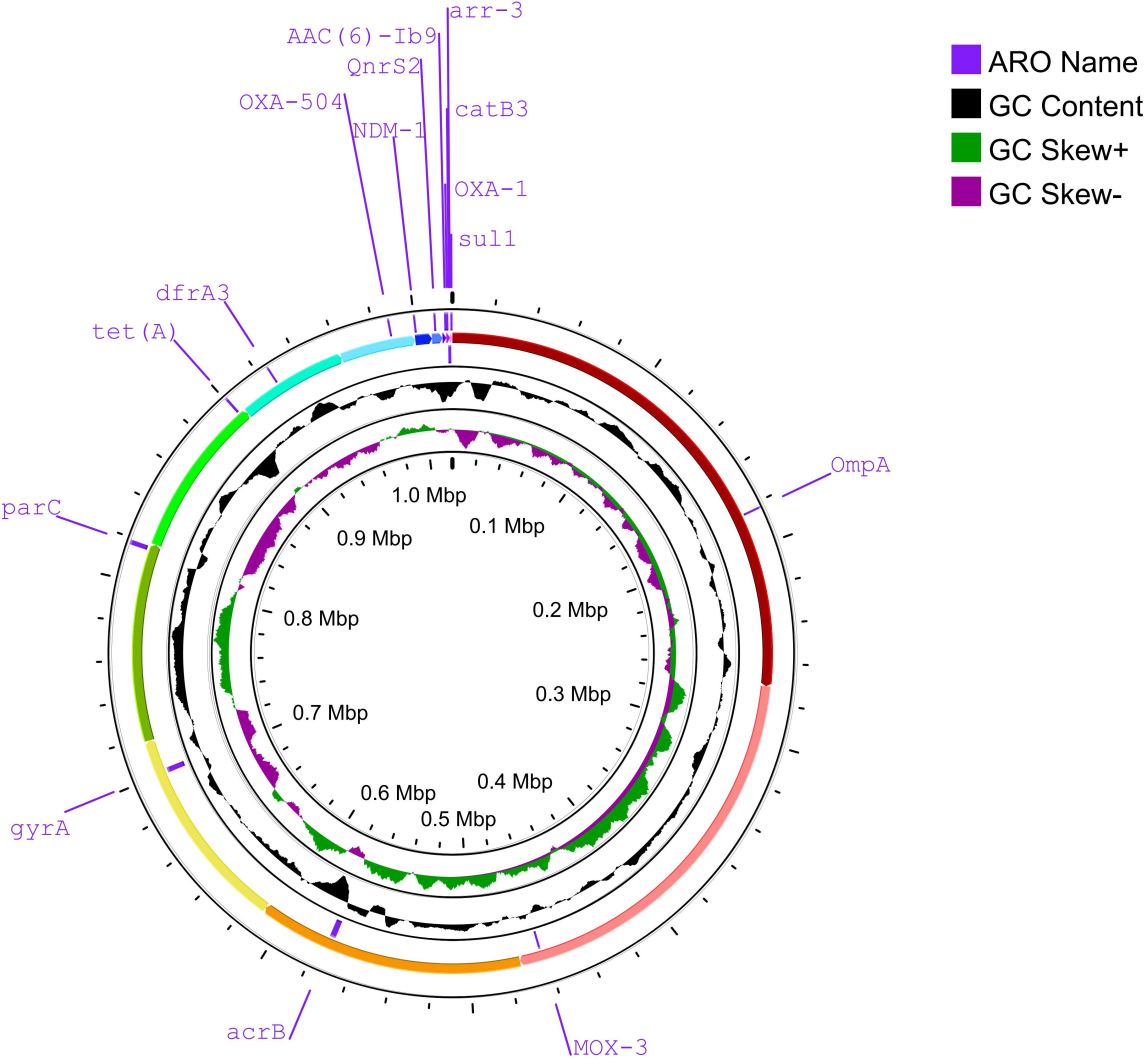
The next-generation sequencing results of Strain 32 are shown in the figure. Key regions of antibiotic resistance and virulence genes were annotated based on BLAST alignment. Genes highlighted in blue-purple indicate resistance genes carried by this strain, including *NDM-1*, *OXA-1*, *catB3*, *dfrA3*, *aar-3*, *acrB*, among others.

Strain 32 was selected as a representative for genomic analysis (**Figure 4**) based on the following criteria. First, its class 1 integron variable region carried the most prevalent resistance gene cassette combination identified in this study. Second, conventional resistance gene screening revealed a comprehensive resistance profile, indicative of the typical multidrug-resistant phenotype observed in *Aeromonas hydrophila*. Genomic sequencing of strain 32 enabled a detailed characterization of its primary resistance mechanisms and provided insights into the broader genetic diversity and genomic complexity of *Aeromonas hydrophila* as a species.

### 3.8 The homogeneity detection results of integron-positive strains

**Figure 5:**
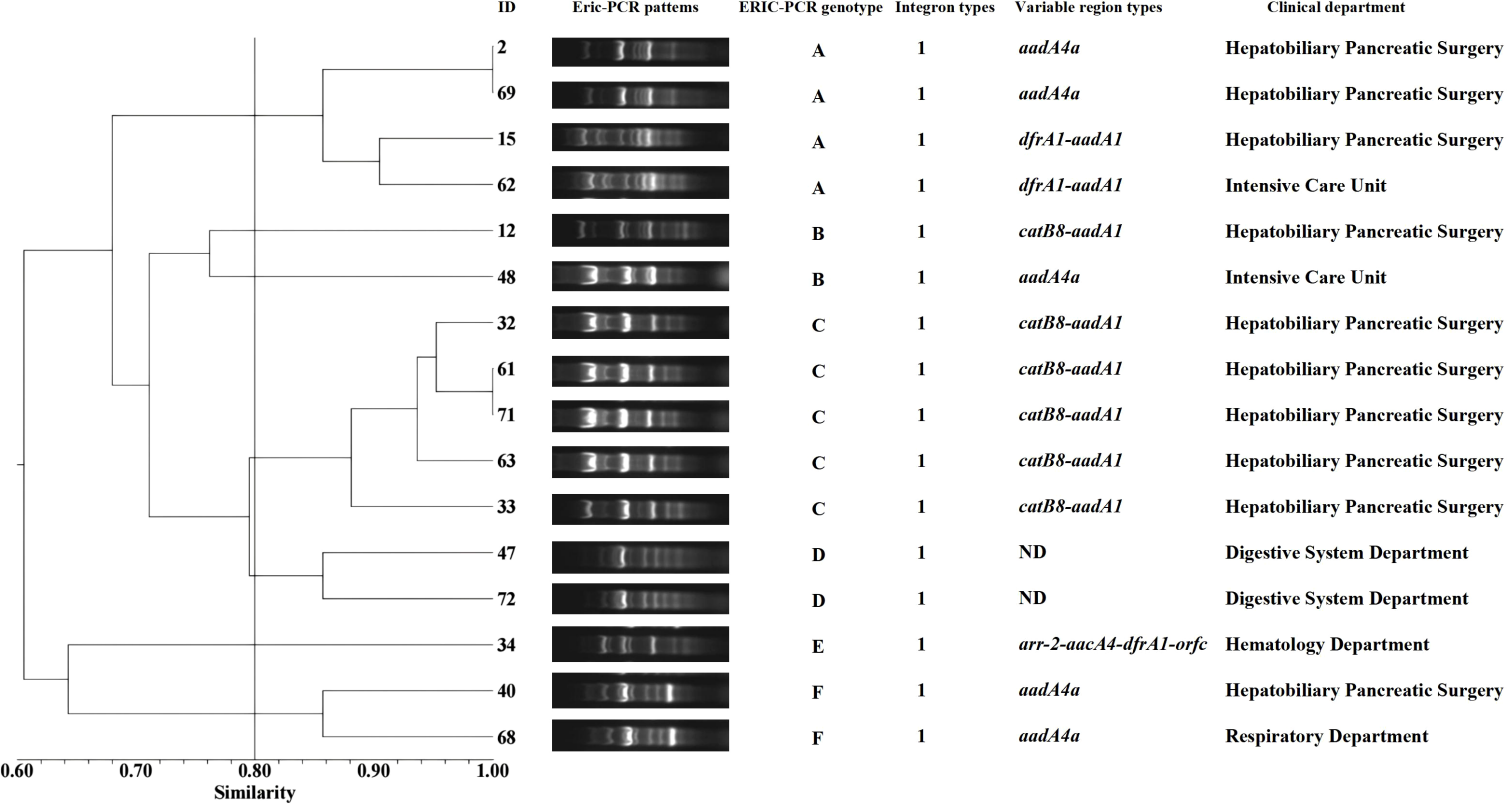
ERIC-PCR genotyping dendrogram of integron-positive strains. Electrophoresis experiments are used to obtain the band positions of different strains. By referring to marker positions, bands at the same location are considered to represent the same genotype, while bands at different positions indicate different genotypes. The Dice similarity coefficient is used to calculate the similarity between strains, which measures the degree of similarity between two strains. The UPGMA method is then applied to transform these similarity data into a phylogenetic tree, ultimately generating a dendrogram that reflects the evolutionary relationships among the strains. ND, Not Detected.

The integron-positive strains were predominantly isolated from the Hepatobiliary and Pancreatic Surgery Department. After ERIC typing, the electrophoretic profiles showed clear amplification bands, with the longest product being approximately 2,000 bp and the shortest below 250 bp. Using a similarity threshold of >80% to define a type, the 16 strains were classified into six ERIC genotypes: type A (25.00%, 4/16), type B (12.50%, 2/16), type C (31.25%, 5/16), type D (12.50%, 2/16), type E (12.50%, 2/16), and type F (6.25%, 1/16). The gene cassette *catB8*–*aadA1*, located in the variable region, was predominantly detected in genotype C. Furthermore, genotype C strains were highly concentrated in the Hepatobiliary and Pancreatic Surgery Department, suggesting possible clonal dissemination within this unit. Details are shown in **Figure 5**.

## 4 Discussion

In recent years, *Aeromonas hydrophila* has gained increasing attention as a significant pathogen in healthcare-associated infections. Not only is it a major causative agent of fulminant septicemia in freshwater fish within the aquaculture industry (Guo et al., 2024), leading to substantial economic losses, but it is also clinically relevant and can cause various human infections including respiratory tract infections, enteric infection, and soft tissue infections (Yamasaki et al., 2020; Vasilev et al., 2021). Particularly in immunocompromised individuals or those with underlying medical conditions, *Aeromonas hydrophila* infection may lead to severe complications and even life-threatening outcomes. However, the resistance of *Aeromonas hydrophila* to commonly used antimicrobial agents, including β-lactams, has been increasing annually, with a high prevalence of multidrug resistance (Sakurai et al., 2023), posing significant challenges to clinical treatment. The spread of multidrug-resistant bacteria within healthcare settings occurs primarily through mobile genetic elements—such as plasmids and transposons—which facilitate the transfer of resistance-related genes via transduction, transformation, and conjugation. These mechanisms play a critical role in the development and dissemination of bacterial antimicrobial resistance. Among these, integrons exhibit a remarkable capacity for the horizontal transfer of antibiotic resistance genes. As mobile genetic elements closely associated with bacterial resistance, integrons can capture and promote the expression of exogenous genes, thereby conferring antimicrobial resistance to their host bacteria (Bhat et al., 2023). Integrons can be located on plasmids or function as components of transposons, thereby facilitating their mobilization and enhancing the dissemination of antibiotic resistance genes. In this study, we analyzed the correlation between integrons/resistance gene cassettes and antimicrobial resistance in 80 *Aeromonas hydrophila* isolates collected from our hospital over the past five years. The dissemination characteristics of integron-positive strains were further evaluated. Consequently, investigating the molecular epidemiology of integrons and their associated resistance genes in clinical Aeromonas hydrophila isolates holds significant scientific and clinical relevance, especially in the context of escalating antimicrobial resistance.

This study first conducted a traceability analysis of 80 clinical *Aeromonas hydrophila* isolates, revealing their distinct clinical distribution patterns. In terms of specimen sources, bile isolates accounted for the highest proportion (35.0%). When combined with their high detection rates in drainage fluid (15.0%) and departments related to the digestive system—Hepatobiliary and Pancreatic Surgery (31.3%), Gastrointestinal Surgery (10.0%), and Gastroenterology (8.8%)—these findings strongly suggest the digestive tract as a major portal of entry for this pathogen (Bhat et al., 2023). This finding is highly consistent with previous epidemiological studies reporting that *Aeromonas hydrophila* can be orally transmitted through contaminated aquatic products or food, further supporting the critical role of foodborne transmission in human infections. Of particular note is the significantly higher isolation rate of this bacterium in patients from the Hepatobiliary and Pancreatic Surgery Department compared to other clinical departments. Further analysis revealed that such patients often present dual predisposing factors, including anatomical abnormalities of the biliary tract (e.g., biliary strictures, sphincter of Oddi dysfunction) and compromised immune function (consistent with other reported studies) (Harandou et al., 2025). Based on these findings, we recommend that: (1) *Aeromonas hydrophila* should be incorporated into the perioperative infection risk assessment protocol for patients undergoing hepatobiliary and pancreatic surgery; (2) Routine screening for *Aeromonas hydrophila* in bile and gut microbiota should be implemented preoperatively for high-risk patients.

This study not only elucidates the clinical distribution and transmission pathways of *Aeromonas hydrophila* but, more importantly, highlights the need for in-depth investigation into its mechanisms of antimicrobial resistance development. As a mobile genetic element, integrons possess a unique structure that enables them to capture and incorporate exogenous genes, transforming them into functional expression units (Fonseca É and Vicente, 2022). They play a critical role in the evolution of antimicrobial resistance in *Aeromonas hydrophila*. Out of the 80 *Aeromonas hydrophila* isolates tested, 16 were found to be positive for class 1 integrons, yielding a positivity rate of 20.0%. This indicates that class 1 integrons are relatively prevalent among these clinical strains. However, the absence of detected class 2 and class 3 integrons may be attributed to their inherently low prevalence in *Aeromonas hydrophila*, though the possibility of methodological limitations cannot be entirely ruled out. However, the detection rate of class 1 integrons in this study was slightly lower than that reported in other Aeromonas species in the environment both domestically and internationally (Otero-Olarra et al., 2020; Dhanapala et al., 2021), which may be associated with regional variations in antibiotic selection pressure specific to *Aeromonas hydrophila* in our clinical setting. Subsequent analysis successfully amplified the variable regions of class 1 integrons in 14 strains, suggesting that *Aeromonas hydrophila* may utilize the integron-borne promoter within this region to regulate the expression of resistance-associated genes (Yang et al., 2024), thereby enhancing its adaptability to diverse environmental stresses and antimicrobial selection pressures. However, no amplification products were observed for the variable regions of class 1 integrons in two strains. This failure may be attributed to potential mutations or sequence deletions (such as 3CS deletion) within the promoter regions of these *Aeromonas hydrophila* isolates (Li et al., 2022). Future studies will consider redesigning primers, optimizing PCR conditions, or employing alternative verification methods to address this issue.

Integrons serve as key vectors for disseminating antibiotic resistance genes among bacteria. Their core function is based on their unique ability to capture, store, and express gene cassettes through site-specific recombination. The specific types of gene cassettes carried by different integrons vary widely, and the resistance genes they encode ultimately determine the bacterial resistance profile to specific antimicrobial agents (Ma et al., 2022). Blast database alignment analysis revealed that four types of resistance gene cassettes were detectable in the amplified variable regions of class 1 integrons in this study. These primarily included genes conferring resistance to chloramphenicol and aminoglycosides, while cassettes encoding resistance to trimethoprim (e.g., *dfrA1*) and rifampin were also identified. This finding provides a genetic explanation for the enhanced resistance to aminoglycosides and trimethoprim observed in integron-positive strains.

Further investigation into the antibiotic resistance and virulence genes of *Aeromonas hydrophila* revealed distinct genetic profiles between clinical and aquaculture-derived strains. While virulence genes such as *act* and *aerA* were commonly detected in aquaculture isolates (Saleh et al., 2021), clinical strains not only carried a distinct set of virulence genes—represented by *ast* (65.0%) and *ahpA* (57.5%)—but also exhibited high prevalence of resistance genes including *MOX* (62.5%) and *ACC* (36.3%). Notably, the carbapenemase gene *NDM-1* was detected in one clinical isolate. These characteristics reflect the strong selective pressures inherent in healthcare environments. Notably, this distinct antimicrobial resistance gene profile is highly consistent with the high resistance rate to ceftazidime observed in clinical isolates. The AmpC enzyme activity mediated by the MOX and ACC genes efficiently hydrolyzes third-generation cephalosporins, representing one of the primary mechanisms leading to ceftazidime treatment failure. However, comparative analysis revealed no significant difference in the carriage of antibiotic resistance genes or virulence genes between integron-positive and integron-negative strains.

Furthermore, this study found that the resistance rates to ceftazidime, gentamicin, imipenem, trimethoprim-sulfamethoxazole, and amikacin were significantly higher in integron-positive strains than in negative strains (*P < 0.05*). Analysis of gene cassettes revealed that the aminoglycoside resistance genes (e.g., *aadA* and *aacA*) and the trimethoprim resistance gene (*dfrA1*) carried by these strains were highly consistent with their phenotypic resistance profiles, providing a molecular-level explanation for the resistance to amikacin and trimethoprim-sulfamethoxazole observed in some isolates. However, the resistance to carbapenems, such as imipenem, could not be fully explained by the detected gene cassettes, suggesting the potential involvement of the following mechanisms: (1) novel resistance gene cassettes within the integron variable region that escaped detection; (2) other resistance genes (e.g., carbapenemase genes such as *KPC*) on plasmids harboring the integrons (Xu et al., 2022); or (3) intrinsic resistance mechanisms mediated by chromosomal alterations, such as porin deficiency or overexpression of efflux pumps (Nayak et al., 2024). These findings not only confirm the central role of the integron-gene cassette system in shaping the resistance phenotypes of *Aeromonas hydrophila*, but also reveal the complex etiology of its resistance—resulting from the combined action of mobile genetic element-mediated acquired resistance and chromosome-mediated intrinsic mechanisms. The molecular epidemiological characteristics elucidated in this study provide critical insights into the transmission and evolution of antimicrobial resistance in this pathogen within the local region.

To comprehensively elucidate the coevolutionary relationships between integron gene cassettes and other mobile genetic elements, and to facilitate the development of targeted antimicrobial resistance containment strategies, strain 32 was selected as a representative isolate for whole-genome sequencing in this study, based on its representative genetic profile and multidrug-resistant phenotype. The genomic sequencing of strain 32 not only confirmed the presence of the resistance gene cassettes (*aadA1* and *catB8*) in the variable region of its class 1 integron, as initially detected by PCR, but also revealed a broader repertoire of resistance and virulence genes that were not captured by conventional PCR-based screening. Key additional resistance genes identified include *gypA*, *parC*, *OXA-504*, and *MOX-3*, among others. These findings demonstrate the utility of whole-genome sequencing in providing a more comprehensive genetic landscape of antimicrobial resistance and virulence.

However, certain discrepancies between the two detection methods were also noted. Although resistance gene cassettes in the variable region of class 1 integrons were successfully detected by PCR, the corresponding sequences were not identified in the next-generation sequencing data. Moreover, the plasmid-borne class 1 integron structure was also missed—a limitation attributable to the inability of the current sequencing strategy to effectively capture plasmid DNA. This discrepancy may result from a combination of the following factors such as plasmid DNA loss during extraction, difficulties in assembling highly repetitive integron regions, and differences in sensitivity between targeted PCR and whole-genome sequencing approaches.

Although *Aeromonas hydrophila* is not among the most common pathogenic bacteria, its transmission in healthcare settings can still lead to infections. Investigating the epidemiological characteristics of this bacterium is crucial for effectively preventing and controlling healthcare-associated infections. Among various epidemiological analysis methods, homology analysis serves as a key tool for elucidating transmission chains and clonal relationships of *Aeromonas hydrophila*. This analysis helps reveal the genetic relatedness among different strains, thereby improving our understanding of their transmission routes and infection characteristics. Based on the ERIC typing results, the *Aeromonas hydrophila* isolates were classified into six distinct genotypes, with type C being the predominant one and primarily distributed in the Hepatobiliary and Pancreatic Surgery Department. This distribution suggests the potential presence of specific strain transmission chains or infection sources within this department, which may be associated with its unique environmental factors, patient population, or medical practices. Such information is of great significance for hospital infection control and prevention, as it can assist healthcare workers in developing targeted strategies to reduce or prevent the transmission and infection of *Aeromonas hydrophila*.

## 5 Conclusion

In conclusion, our study reveals that class 1 integrons are the predominant type carried by *Aeromonas hydrophila* clinical isolates in our hospital. The aminoglycoside resistance gene *aadA1* and the chloramphenicol resistance gene *catB8*, identified within the variable regions of these integrons, are directly associated with corresponding antibiotic resistance phenotypes. Notably, integron-positive strains displayed high clonal similarity, with the dominant ERIC genotype C indicating potential clonal dissemination within the hospital setting. These findings suggest that integrons, along with their carried resistance gene cassettes, could serve as useful molecular markers for epidemiological surveillance of multidrug-resistant *Aeromonas hydrophila*. Implementing routine screening for integrons and associated resistance genes in high-risk wards, such as hepatobiliary surgery, could enhance targeted infection control measures and help prevent the spread of resistant clones, including those of emerging pathogens like *A. hydrophila*.

## Data Availability

The datasets presented in this study can be found in online repositories. The names of the repository/repositories and accession number(s) can be found in the article/**Supplementary Material**.
