# Supplementary material for: Distribution characteristics of integrons and correlation analysis of antibiotic resistance in Aeromonas hydrophila
Source: Front Cell Infect Microbiol. 2026 Jan 16;15:1709447. doi: 10.3389/fcimb.2025.1709447 (PMC12855559; doi:10.3389/fcimb.2025.1709447)
Supplement: Supplementary file 1 [file Table1.docx]

**Supplemental material**

**Supplementary Table1** Primers used for PCR amplification

| **Primer** | **Primer sequence(5＇-3＇)** | **Product**  **（bp）** |
| --- | --- | --- |
| *intF* | CCAAGCTCTCGGGTAACATC | 615 |
| *P2R* | CCCGAGGCATAGACTGTA |  |
| *intI2F* | GTAGCAAACGAGTGACGAAATG | 788 |
| *intI2R* | CACGGATATGCGACAAAAAGGT |  |
| *intI3F* | AGTGGGTGGCGAATGAGTG | 615 |
| *IntI3R* | TGTTCTTGTATCGGCAGGTG |  |
| *5CS* | GGCATCCAAGCAGCAAG | / |
| *3CS* | AAGCAGACTTGACCTGA |  |
| *INF2* | TGGGTGAGATAATGTGCATC | / |
| *INB2* | TCGAGAGAGGATATGGAAGG |  |
| *CTXM-1-F* | ATGGTTAAAAAATCACTGCGYCAGTTC | 876 |
| *CTXM-1-R* | TCACAAACCGTYGGTGACGATTTTAGCCG |  |
| *CTXM-2-F* | ATGATGACGCAGAGCATTCGCCGCTCA | 876 |
| *CTXM-2-R* | TCAGAAACCGTGGGTTACGATTTTCGC |  |
| *CTXM-8-F* | ATGATGAGACATCGCGTTAAGCGG | 876 |
| *CTXM-8-R* | TTAATAACCGTCGGTGACGATTTTCGCG |  |
| *CTXM-9-F* | ATGGTGACAAAGAGAGTGCAACGG | 876 |
| *CTXM-9-R* | TTACAGCCCTTCCCCGATGATTCTCGC |  |
| *CTXM-25-F* | ATGATGAGAAAAAGCGTAAGGCGGGCG | 876 |
| *CTXM-25-R* | TTAATAACCGTCGGTGACAATTCTGGC |  |
| *TEM-F* | AGGAAGAGTATGATTCAACA | 535 |
| *TEM-R* | CTCGTCGTTTGGTATGGC |  |
| *SHV-F* | TCCGCAAGCTGCTGACCAGC | 305 |
| *SHV-R* | TTAGCGYTGCCAGTGCTCGA |  |
| *PER-F* | AGTCAGCGGCTTAGATA | 978 |
| *PER-R* | CGTATGAAAAGGACAATC |  |
| *GES-F* | ATGCGCTTCATTCACGCAC | 846 |
| *GES-R* | CTATTTGTCCGTGCTCAGG |  |
| *VEB-F* | GCGGTAATTTAACCAGA | 961 |
| *VEB-R* | GCCTATGAGCCAGTGTT |  |
| *LAP-F* | ATGAAAAAGATCCGCCTTATTATAA | 858 |
| *LAP-R* | TTACCAGTTCTTAATTACTGAATC |  |
| *AQU-F* | ATGAAGCAAACCTCACCCTTG | 1143 |
| *AQU-R* | TCAGGGAGCCCAGCTTGCTCAG |  |
| *DHA-F* | AACTTTCACAGGTGTGCTGGGT | 405 |

**Supplementary Table1（Cont）**

| **Primer** | **Primer sequence(5＇-3＇)** | **Product**  **（bp）** |
| --- | --- | --- |
| *DHA-R* | CCGTACGCATACTGGCTTTGC |  |
| *ACT-F* | TCCGTAAAGCCGATGTTGCG | 302 |
| *ACT-R* | CTTCCACTGCGGCTGCCAGT |  |
| *LAT-F* | TGGCCAGAACTGACAGGCAA | 462 |
| *LAT-R* | TTTCTCCTGAACGTGGCTGG |  |
| *MOX-F* | GCTGCTCAAGGAGCACAGGAT | 520 |
| *MOX-R* | CACATTGACATAGGTGTGGTG |  |
| *ACC-F* | ACAGCCTCAGCAGCCGGTTA | 345 |
| *ACC-R* | TTCGCCGCAATCATCCCTAG |  |
| *FOX-F* | AACATGGGGTATCAGGGAGAT | 190 |
| *FOX-R* | CAAAGCGCGTAACCGGATTGG |  |
| *OXA-1-F* | CTCTTGTTTGGGTTTCGCAAG | 440 |
| *OXA-1-R* | CTTGGCTTTTATGCTTGATG |  |
| *OXA-2-F* | CAGGCGCYGTTCGYGATGAGTT | 233 |
| *OXA-2-R* | GCCYTCTATCCAGTAATCGCC |  |
| *OXA-10-F* | GTCTTTCRAGTACGGCATTA | 822 |
| *OXA-10-R* | GATTTTCTTAGCGGCAACTTA |  |
| *NDM-1-F* | GGTTTGGCGATCTGGTTTTC | 621 |
| *NDM-1-R* | CGGAATGGCTCATCACGATC |  |
| *SUL1-F* | TAGCGAGGGCTTTACTAAGC | 300 |
| *SUL1-R* | ATTCAGAATGCCGAACACCG |  |
| *dfrA1-F* | TTGTGAAACTATCACTAATGGTAG | 480 |
| *dfrA1-R* | CTTGTTAACCCTTTTGCCAGA |  |
| *dfrA12-F* | ATGAACTCGGAATCAGTACGC | 498 |
| *dfrA12-R* | TTAGCCGTTTCGACGCGCAT |  |
| *dfrA17-F* | TTGAAAATWTCATTGATTTCT | 474 |
| *dfrA17-R* | TTAGCCTTTTTTCCAAATCTGRTATGT |  |
| *aerA-F* | CAAGAACAAGTTCAAGTGGCCA | 309 |
| *aerA-R* | ACGAAGGTGTGGTTCCAGT |  |
| *hlyA-F* | TGACAGGCAAGTAGAATAACGC | 1815 |
| *hlyA-R* | TGTCCGCTTTCCACTCCC |  |
| *ahpA-F* | GTTAGCGTTGGCAATCTCG | 874 |
| *ahpA-R* | CGCTGGAGTAGGAGGAACG |  |
| *ast-F* | TGACCCAGTCCTGGCACGGC | 504 |
| *ast-R* | GGTGATCGATCACCACCAGC |  |
| *altA-F* | ATCGTCAGCGACAGCTTCTT | 442 |
| *altA-R* | CTCATCCCTTGGCTTGTTGT |  |
| *ERIC2* | AAGTAAGTGACTGGGGTGAGCG | / |
